# Guillain–Barré syndrome following different COVID-19 vaccines: a case series

**DOI:** 10.1186/s41983-022-00582-7

**Published:** 2022-12-09

**Authors:** Ali Shalash, Nourhan Belal, Amr S. Zaki, Shady S. Georgy, Mohamed Fahmy Doheim, Ahmed Hazzou, Azza Abdelnasser

**Affiliations:** 1 Department of Neurology, Faculty of Medicine, Ain Shams University, 168 Elnozha St, Saint Fatima Square, Heliopolis, Cairo, Egypt; 2 Faculty of Medicine, Alexandria University, Alexandria, Egypt

**Keywords:** Guillain–Barré syndrome, COVID-19, Vaccines, Case series

## Abstract

**Background:**

The COVID-19 vaccine-related Guillain–Barré syndrome (GBS) has been described for both messenger-RNA vaccine and adenovirus-vectored types in a few cases with great public concern and the necessity to inform physicians about the variations of its presentations given its life-threatening consequences.

**Case presentation:**

This case series highlighted the presentation with GBS following different COVID-19 vaccinations in seven cases with ages ranging from 29 to 59 years. Three patients received the AstraZeneca vaccine, two received the Pfizer vaccine, one received Sinopharm, and one received the Janssen vaccine. Latency ranged from 5 to 60 days and cases achieved either partial or complete improvement after treatment trials. Patients responded to plasmaphereses, but not pulse steroid therapy.

**Conclusion:**

This case series highlights the variable presentations and outcomes of GBS following different COVID-19 vaccination from one center. The early identification and appropriate management of such cases can lead to better outcomes.

## Background

Guillain–Barré syndrome (GBS) is an immune-mediated polyradiculoneuropathy that is usually preceded commonly by infections such as *Campylobacter jejuni*, Mycoplasma pneumonia, the alphavirus chikungunya, the flaviviruses and viral outbreaks (Zika virus) [1]. Moreover, it has been linked to vaccination such as the influenza vaccine[2]. With the emergence of severe acute respiratory syndrome coronavirus 2 (SARS-CoV-2) infection, the association of GBS has been increasingly reported worldwide [3, 4].

More recently, COVID-19 vaccine-related GBS has been described for both messenger-RNA vaccine and adenovirus-vectored types in a few cases [5, 6]. Although it is relatively uncommon, identifying the clinical presentations and the outcome of GBS following different COVID-19 vaccinations among different populations is essential, given the life-threatening consequences.

Therefore, we report a case series of seven patients who presented to Ain Shams hospitals from October 2021 to February 2022 by GBS following different COVID-19 vaccinations, describing their clinical manifestations, response to treatment and outcome. Patients were assessed pre- and post-treatment using the GBS Disability Score [7, 8]. Written consent was obtained from all patients, and we followed the CARE guidelines for reporting those cases.

## Case presentations

A summary of all cases that developed GBS following COVID-19 vaccination is provided in Table 1.

**Table 1:** Summary of the cases presented with Guillain–Barré syndrome following COVID-19 vaccination

Cases	1	2	3	4	5	6	7
Gender	Male	Male	Male	Male	Male	Male	Female
Age	39 years	29 years	59 years	53 years	55 years	59 years	29 years
Past history	COVID-19 vaccination (2nd dose)	COVID-19 vaccination (2nd dose)	COVID-19 vaccination (2nd dose)	Ischemic heart disease, COVID-19 vaccination (2nd dose)	COVID-19 vaccination (2nd dose)	Diabetes mellitus, hypertension, ischemic heart disease COVID-19 vaccination (3rd dose)	Portal and splenic vein thrombosis two months after receiving COVID 19 vaccination (2nd dose)
Type of vaccine	Pfizer	Janssen	AstraZeneca	AstraZeneca	Pfizer	Sinopharm	AstraZeneca
Latency	5 days	20 days	30 days	14 days	30 days	30 days	60 days
Presentation	Bilateral upper eyelid partial ptosis, bilateral ULs and LLS tingling and numbness, bilateral LLs and ULs flaccid weakness (proximal more than distal), truncal weakness, areflexia, bilateral glove and stocking hypesthesia	Bilateral ULs and LLs tingling and numbness, bilateral LLs and ULs flaccid weakness (proximal more than distal), areflexia, stretch signs, left facial nerve weakness	Bilateral ULs and LLs tingling and numbness, bilateral LLs flaccid weakness (proximal more than distal), bilateral ULs weakness (distal more than proximal), areflexia, stretch signs	Bilateral ULs and LLs tingling and numbness, bilateral ULs and LLs flaccid weakness (proximal more than distal), truncal muscle weakness, areflexia and respiratory muscle weakness	Low back pain, bilateral LLs tingling and numbness, bilateral LLs flaccid weakness (proximal more than distal), areflexia	Tingling in both LLs then ULS and bilateral LLs and ULs flaccid weakness (proximal more than distal) then bilateral facial nerve weakness, areflexia	Bilateral LLs tingling and numbness followed by bilateral ULs tingling and numbness then developed bilateral ULs and LLs flaccid weakness more on left, areflexia and bilateral facial nerve weakness,
NCV	Sensory and motor axonal neuropathy	Axonal polyneuropathy of both lower limbs, with proximal neurogenic affection,	bilateral axonal polyradiculoneuropathy of both lower limbs	bilateral axonal polyradiculoneuropathy of LLs	bilateral axonal polyradiculoneuropathy of LLs	NA	Mixed axonal and demyelinating polyradiculoneuropathy
CSF	Protein 38 mg/dl, glucose 93 mg/dl, LDH 15 u/l, no cells	NA	NA	NA	Protein 40.4 mg/dl, glucose 122.1 mg/dl, LDH 18.6 u/l, no cells	Protein 112 mg/dl, glucose 70 mg/dl and no cells	NA
Treatment	8 plasmapheresis sessions	Daily intravenous methylprednisolone 1 g for 7 days and 9 plasmapheresis sessions	Daily I intravenous methylprednisolone 1 g for 7 days and 6 plasmapheresis sessions	Daily intravenous methylprednisolone 1 g for 7 days and 6 plasmapheresis sessions	Daily intravenous methylprednisolone 1 g for 7 days then 7 plasmapheresis sessions	5 sessions of plasmapheresis	7 plasmapheresis and arrested in the 7th session then regained, stopped sessions, and received 6 doses of methyl prednisolone with no improvement and discharged on prednisone 60 mg
Guillain–Barré syndrome (GBS) Disability Score (pretreatment)	4	4	4	5	4	4	4
Guillain–Barré syndrome Disability Score (post-treatment)	0	1	1	4	0	1	4
Outcome	Complete improvement	Partial improvement	Complete improvement except residual numbness	Partial improvement	Complete improvement	Complete improvement as regard weakness but still has tingling sensation in both upper and lower limbs	Partial improvement with regard to upper limb weakness

*LL* lower limbs, *ULs* upper limbs, *NCS* nerve conduction study, *CSF* cerebrospinal fluid, *NA* not available

### Case 1

A 39-year-old male patient presented 5 days after receiving COVID-19 vaccination (BNT162b2 vaccine, Pfizer–BioNTech) with bilateral upper eyelid partial ptosis, quadriparesis, truncal muscle weakness, bilateral upper and lower limbs tingling and numbness, and bilateral glove and stocking hypesthesia. A nerve conduction study (NCS) showed sensory and motor axonal neuropathy. Cerebrospinal (CSF) analysis was performed with no remarkable findings (Protein 39 mg/dl, glucose 93 mg/dl, LDH 15 u/l and no cells). The patient received 8 plasmapheresis sessions and showed complete improvement.

### Case 2

A 29-year-old male patient presented 20 days after receiving COVID-19 vaccination on 11/11/2021 (Ad26.COV2.S, Janssen) with bilateral upper and lower limb tingling and numbness, quadriparesis, radicular pain and left lower motor neuron lesion of the facial nerve. A nerve conduction study showed axonal polyneuropathy of both lower limbs. The patient received daily intravenous methylprednisolone 1 g for 7 days followed by 9 plasmapheresis sessions and showed complete improvement.

### Case 3

A 59-year-old male patient presented 30 days after receiving COVID-19 vaccination (ChAdOx1 nCoV-19, AZD1222, Oxford–AstraZeneca) with bilateral upper and lower limb tingling and numbness, quadriparesis and radicular pain. The NCS showed bilateral axonal polyneuropathy of both lower limbs. The patient received daily intravenous methylprednisolone 1 g for 7 days followed by 6 plasmapheresis sessions with complete improvement.

### Case 4

A 53-year-old male patient, with history of ischemic heart disease, presented 14 days after receiving COVID-19 vaccination (ChAdOx1 nCoV-19, AZD1222, Oxford–AstraZeneca) with bilateral upper and lower limb numbness, quadriparesis, truncal muscle weakness, lower limbs radicular pain, and respiratory muscle weakness. The patient was admitted to ICU and required continuous positive airway pressure (CPAP). The NCS showed bilateral axonal polyradiculoneuropathy of both lower limbs. The patient received 12 plasmapheresis sessions and daily intravenous methylprednisolone 1 g for 7 days and showed partial improvement regarding the respiratory muscle weakness and came off CPAP after the first 3 successive sessions with no improvement of limbs’ weakness and numbness. The patient was discharged and died one month later.

### Case 5

A 55-year-old male with a history of COVID-19 infection, presented 30 days after receiving COVID-19 vaccination (BNT162b2 vaccine, Pfizer–BioNTech) with low back pain, bilateral lower limbs numbness and weakness (proximal more than distal). The NCS was done and showed bilateral axonal polyradiculoneuropathy of both lower limbs, CSF analysis showed (protein 40.4 mg/dl, glucose 122.1 mg/dl, LDH 18.6 u/l and no cells). The patient received daily intravenous methylprednisolone 1 g for 7 days and 7 plasmapheresis sessions with complete improvement of the neurological symptoms. However, the patients developed fever and PCR for COVID 19 came positive, so the patient was isolated in ICU. Few days later, the patient developed acute kidney injury (serum creatine 10 mg/dl) and received dialysis sessions.

### Case 6

A 59-year-old male with a history of diabetes, hypertension, and ischemic heart disease, presented 30 days after receiving COVID 19 vaccination (3rd dose of Sinopharm vaccine on 1/2022), with tingling in both lower then upper limbs and bilateral lower and upper limb weakness (proximal more than distal) to the degree that patient became bilaterally supported, and areflexia followed by bilateral facial weakness. The CSF analysis showed high protein (112 mg/dl), average glucose (70 mg/dl) and no cells. The patient received 5 sessions of plasmapheresis and showed complete improvement except for mild numbness.

### Case 7

A 29-year-old female presented with a history of portal and splenic vein thrombosis 2 months after receiving COVID 19 vaccination (Astrazeneca on 21/5/2021 and 20/8/2021). Two months after the last dose, the patient developed bilateral lower limbs tingling and numbness followed by bilateral upper limbs tingling and numbness followed by bilateral upper and lower limb weakness more on the left side, areflexia, and bilateral facial nerve weakness. The NCS and Electromyography (EMG) showed mixed axonal and demyelinating polyradiculoneuropathy. The patient received 7 sessions of plasmaphereses, but cardiac arrest occurred during the 7th session with rapid regain of normal function and consciousness. The patient did not undergo further sessions and received 6 doses of methylprednisolone with partial improvement with regard to upper limb weakness and was discharged on oral prednisone.

## Discussion

In this case series, six males and one female with ages ranging from 29 to 59 years were presented with variable GBS manifestations from December 2021 to February 2022. Three patients received the AstraZeneca vaccine, two received the Pfizer vaccine, one received the Sinopharm and one received Janssen vaccine. Latency ranged from 5 to 60 days and cases achieved either partial or complete improvement after plasmapheresis. In a review of 19 cases with GBS post-vaccination, there was a high variability in the latency between vaccination and onset of GBS following COVID-19 vaccinations and mild to severe complications with six patients requiring mechanical ventilation. Although the outcome was favorable, partial recovery only was achieved [6]. For instance, four patients presented with bifacial muscle weakness and paresthesia after administering vector-based SARS-CoV-2 vaccine with a latency of three weeks that was classified as polyneuritis cranialis variant of GBS. Among them, three patients received intravenous immunoglobulins or steroids, one patient recovered without treatment and none of them required mechanical ventilation [5]. Another reported 32 year-old male developed acute inflammatory demyelinating polyneuropathy (AIDP), with a latency of 8 days from the first dose of a vector-based SARS-CoV-2 vaccine [9]. He had a previous history of AIDP 14 years earlier and recovered completely via two intravenous immunoglobulin (IVIG) cycles and plasmapheresis but is still handicapped undergoing immune adsorption [9]. A 60-year-old female patient also developed back and leg pain followed by headache, double vision, nausea, vomiting, facial diplegia, and paraparesis of the lower limbs. She benefitted from intravenous immunoglobulins and partially recovered [8]. Likewise, seven other patients were reported after administering a vector-based SARS-CoV-2 vaccine with cranial nerve involvement [10]. The NCS showed the absence of late responses and was interpreted as early signs of GBS. In a recent case series, two older female patients in remission from diffuse large B-cell lymphoma developed GBS; with subtypes including acute motor and sensory axonal neuropathy and acute inflammatory demyelinating polyneuropathy after receiving the Pfizer vaccine [11].

In those aforementioned cases, GBS occurred following the vaccination. The vaccinations lead to producing T cells and antibodies and this could cross-react with the nerve roots structures [12]. Facial paralysis (FP) could be seen in cases with vaccine-related GBS. In a study by Pegat et al., they showed cases reported from the French pharmacovigilance database and among the 48,907 cases with COVID-19 vaccines, there were 69 cases of GBS, of which 23 involved FP. This included 2 of 22 GBS cases who received mRNA vaccines namely Pfizer–BioNTech and 21 of 47 who received adenovirus-vectored vaccines (20 of 44 Oxford–AstraZeneca, 1of 3 Johnson & Johnson), which showed a high frequency of FP-GBS occurring following adenovirus-vectored vaccines [13].

Moreover, five cases of bilateral facial weakness with paresthesia variant of GBS COVID-19 vaccine [14] while three patients who developed GBS following ChAdOx1 nCoV-19 vaccination were reported with a latency of 11–13 days after the first dose of vaccine. All had sensorimotor weakness of both the upper and lower limbs and facial diplegia in one and dysautonomia in the other. The NCSs were supporting demyelination in two and axonopathy in one. In addition, CSF analysis revealed albuminocytological dissociation in two patients. They were treated with intravenous immunoglobulin, resulting in stabilizing of the disease [15]. The current report confirms the favorable response of post-COVID-19 immunization to plasmapheresis, rather than IV methylprednisolone. Recent studies reported more frequent use of IVIG, with less frequent use of plasmapheresis and rare use of steroids [16, 17]. Similar to thesis reports, plasmaphereses showed favorable outcome [16, 17]. Plasmapheresis was used as it is more affordable and available.

Limitations of this report include not performing CSF analysis, but the diagnosis was confirmed clinically and electrophysiologically. Remarkably, the cases were presented to a referral specialized neurological department and were assessed by experts.

## Conclusion

This case series highlights the variable presentations and the outcome of seven cases with GBS following different COVID-19 vaccination from one center. We draw attention to such cases as early identification and appropriate management can lead to better outcomes.

## Data Availability

All data generated or analyzed during the current study are included in this published article.

## Abbreviations

GBS: Guillain–Barré syndrome
RNA: Ribonucleic acid
SARS COV 2: Severe acute respiratory syndrome corona virus 2
CSF: Cerebrospinal fluid
NCS: Nerve conduction study
CPAP: Continuous positive airway pressure
ICU: Intensive care unit
EMG: Electromyography
AIDP: Acute inflammatory demyelinating polyneuropathy
IVIG: Intravenous immunoglobulin
FP: Facial paralysis
M RNA: Messenger ribonucleic acid
